# Audit of operational noise at the leicester royal infirmary aicu

**DOI:** 10.1186/2197-425X-3-S1-A146

**Published:** 2015-10-01

**Authors:** M Charlton, KA Prosho, D O'Neil

**Affiliations:** Leicester Royal Infirmary, Department of Anaesthesia and Critical Care, Leicester, United Kingdom; Leicester Royal Infirmary, Adult Intensive Care Unit, Leicester, United Kingdom

## Introduction

Ambient noise and its impact on sleep is an important factor in the development of delirium[1]. Noise is a problem on intensive care units, being one of the main environmental stress inducing factors amongst patients[2]. A key source of noise is the signalling of alarms.

As part of plans to decrease noise, we investigated the number and appropriateness of alarms sounded overnight. An initial audit demonstrated that over 53% of alarms were inappropriate, with two recurring sources being due to sampling from indwelling lines and physiological variables falling outside of a pre-programmed range.

Following this we implemented a brief educational programme, highlighting the importance of setting appropriate alarm limits and muting prior to sampling from lines. We then re-audited to determine if improvement had been made.

## Objectives

Improvement in the frequency of inappropriately sounded alarms at night following intervention.

Audit standard: 90% of alarms being deemed as appropriate.

## Methods

Data was collected prospectively by nursing staff on the intensive care unit. Nurses were allocated to collect data on a bed-space other than their own to reduce bias. Data collected included the source and response to the alarm. Data was reviewed by the authors to determine if the alarms and response were appropriate.

## Results

Data was collected over 7 nights with the total number of recorded alarms being 200. Alarm sources included patient monitoring, infusion pumps and ventilators amongst others. 39% of the alarms were deemed to be inappropriate with 7.7% of these being due to sampling from arterial lines. Other frequently occurring alarms included physiological variables and minute/tidal volumes falling outside of pre-determined ranges (34.6% and 15.4% respectively).

## Conclusions

Compared to the first audit, the number of alarms deemed as inappropriate has reduced from 53.9% to 39%. Alarms due to sampling from indwelling lines have reduced from 20.8% to 7.7%. Alarms due to physiological variables falling outside of the pre-determined ranges have increased from 27.1% to 34.6%.

Ensuring quality sleep forms an integral part of a patient's recovery from critical illness and its lack thereof plays an important role in the development of delirium with its subsequent effects on morbidity and mortality. Excess ambient noise due to inappropriate alarms will impact on sleep.

Despite some improvement, we have failed to meet our self-imposed audit standard. We plan to reaffirm the muting of alarms prior to taking blood samples and reinforce the importance of appropriate setting of physiological variable limits at the start of each clinical shift. We plan to implement a daily checklist to ensure this is complete.
